# Clinical Report on Chronic Pleurisy, Based on the Analysis of 47 Cases

**Published:** 1854-03

**Authors:** 


*Clinical Report on Chronic*]*Pleurisy, Based on the Analysis of* 47 *Cases*; by Austin Flint, M. D., Prof. of Principles and Practi·e of Medicine in the University of Buffalo, N. Y., and in the University of Louisville, Ky.

*Clinical Report on Dysentery*. Based on Analysis of 49 Cases; with Remarks on the Causation, Pathology and Management of the Disease; by Austin Flint, M. D., Prof. &c., &c. Buffalo: printed by Jewett, Thomas, & Co, 1853.

The first-named of the above Reports is a monograph of 58 pages. The cases which constitute the basis of the work, are thus described by the author :

" A considerable proportion of the patients were either seen in consultation, or applied simply for a physical examination of the chest. Under these circumstances, a general sketch of the previous history only was noted, and in several instances the farther progress and issue were not procured. The cases, moreover, came under my notice at different stages of the history, and the notes of some were made after recovery from the disease. The data which I have collected are, thus, manifestly inadequate for the positive and negative results of a complete numerical analysis. To this character the present report can have no claim. My only expectation is to present a small contribution towards the cultivation of a field of clinical study of great practical interest, which, from the difficulty of aggregating facts, or other causes, has hitherto been neglected. The number of cases, of which I have preserved notes, is *forty-seven.* These have occurred, some in hospital and others in private practice, for the most part during the last eighteen years. A few are of an older date. For convenience of consideration and reference, they admit of being distributed into several groups. One of these groups will embrace the cases which came under observation while the disease was progressing either favorably or unfavorably. Another group will include cases in which the diagnosis and history were retrospective, that is to say the facts were obtained at a period more or less distant from the occurrence of the disease, being restricted in a great measure to its consequences or sequelæ. Another will embrace cases of circumscribed, as distinguished from general pleurisy. Another will embrace cases of pleurisy from perforation, constituting the affection known by the incorrect title of pneumo-hydro-thorax. I shall speak of these several groups, excepting the first, under distinct heads, but with reference to some points of inquiry it will be proper to examine the cases collectively, disregarding the foregoing divisions."

From this, and still more from a perusal of the subsequent pages of the work, it will be seen that only a part of the cases included in the forty-seven were actually kept under observation, and the phenomena recorded in such a manner, as to make them valuable for analysis.

One of the greatest defects in the present habits of medical practitioners is the neglect to record from day to day full notes of the more important cases of disease occurring under their observation.—Our literature is filled with mere statements from memory—loose generalizations—many of which would be essentially modified by a comparison with an extensive and detailed record of cases, made *while the cases were present* under the eye of the observer. I know it is alleged that the active practitioner has no time to make such a record. And in many instances the allegation is true if an attempt is made to record, at the same time, all the cases of every kind of disease that the physician meets with. But I believe every physician, however large may be his practice, *can, if he will*, keep a detailed and reliable record of all the cases of any *one* disease that he may meet with. And if this were done in relation to one disease for *one* year, and another the next, and so on, he would not fail to accumulate in a few years a most valuable collection of facts for comparison and deduction. In respect to fullness in the recorded history of cases, I confess to have been disappointed in the monograph of Dr. Flint. Neither do I find in looking over its pages somewhat carefully, any important additions to our previous knowledge of the pathology and treatment of chronic pleurisy. Still the essay is well written and valuable; and should be found in every physician's library.—The truth of the following paragraph in reference to diagnosis should excite in the minds of the profession more careful attention to this subject:—

The number of cases in which errors of diagnosis are noted to have occurred, is eighteen. Of these eighteen cases, in four the disease had been treated as latent intermittent fever. This error was based, in two instances, on the fact that chills were pretty prominent, and in the other cases on the fact that the patients had been exposed to miasmata. In four cases the disease was supposed to be phthisis. In two cases the patients were thought to be laboring under continued fever, and in one case the disease was developed in the course of fever and overlooked. In the remaining seven cases, the supposed affection was in each case different. The following is a list of the diagnostic views that had been entertained: Disease of the heart, abscess between the pleura and walls of chest, bilious fever, hepatization of lung, liver complaint, general debility, and some pulmonary affection the nature of which was confessedly not known.

The diagnosis of Chronic Pleurisy, with the aid of physical exploration, is as simple and sure, as it is difficult and doubtful when the sole dependence is on the symptoms. Flatness on percussion extending from the bottom of the chest, upward, over the whole, or a considerable portion of one side, anteriorly, posteriorly, and laterally, and absence of respiratory sound, would alone lead to a correct diagnosis, with some very rare exceptions. A tumor filling the chest on one side, in part, or entirely, constitutes almost the only morbid condition in which the above combination of signs is presented, exclusive of liquid effusion. Solidified lung rarely, if ever, occasions the same absolute loss of sonoriety, in the same situation, and to the same extent, coupled with absence of all respiratory sound; and, on the other hand, the presence of a bronchial respiration in cases of large liquid effusion is an anomaly of which but a few instances are on record. But aside from the above combination, if the liquid effusion be large, as it generally is in Chronic Pleurisy. we have the enlargement of the chest on the affected side, its comparative immobility, the widening and elevation of the intercostal spaces, the displacement of the heart if the affection be seated in the left side, the contraction of the affected side after the liquid effusion has been removed wholly, or in part, by absorption, the change of level of the fluid if the quantity be moderate and the pleural surfaces free, a friction sound in some cases, the absence of the vocal fremitus frequently appreciable on the sound side, and occasionally the œgophonous modification of bronchophony. With the assistance of more or less of these collateral signs, the discrimination is easy and certain. Reference is now made to Chronic *general* Pleurisy. Circumscribed pleurisy may involve difficulties in the way of diagnosis to which it will be more appropriate to refer in another connection. To treat of the diagnosis at any length, does not fall within the scope of this Report. After indulging, however, inthe few foregoing remarks, I may briefly allude to one or two points of practical interest which are incidentally connected with the subject.

The second paper whose title has been given above contains 90 pages, and consists of two parts.

The first part contains a statistical or rather numerical analysis of 49 cases of dysentery, occurring in the city of Buffalo, N. Y., during a series of years reaching from 1838 to 1853. The second part is in the form of a Supplement, and contains a brief account of the Morbid Anatomy of Dysentery as given by Rokitanski, with remarks on the causation, pathology, and management of the disease.—The following quotation will give the reader a correct idea of the materials and arrangement of the first division :—

Among the notes that I have collected of diseases occurring in private and hospital practice, are embraced histories, more or less complete, of sixty cases of dysentery. On examination of these cases with a view to analysis, eleven were rejected in consequence of the patients having come under observation quite late in the progress of the disease, and the records being very imperfect. In two of these rejected cases the isue was fatal ; in the others, either recovery took place, or the disease continued when the patient passed from under my observation. The residue of cases, after this elimination, amounted to forty-nine. These cases I have subjected to analysis. Taking up the several histories in succession, every thing noted in each history relating to the objects of inquiry contained in a series of sections, has been selected and arranged, each in the particular section to which it appropriately belongs. The sections are as follows :

1. Name. Age. Sex. Occupation. Season and year. Previous health and Constitution. Duration of disease before coming under observation.
2. Circumstances attending the access of the disease.
3. Symptoms referable to the Digestive System.
4. Symptoms referable to the Circulatory System.
5. Symptoms referable to the Nervous System.
6. Symptoms referable to the Skin.
7. Symptoms referable to the Respiratory System.
8. Duration of disease to death or convalescence. Mode of dying. Relapses. Fatality.
9. Supposed causative agencies.
10. Subsequent health. Recurrence of the disease.
11. Treatment, and immediate (apparent) effect of remedies.

It would appear from the analysis of Dr. Flint that age, and season of the year, both exerted a decided influence over the prevalence of dysentery. Thus of the 44 cases in which the age was noted, 30 occurred between the ages of 19 and 33 years. And of the same number of cases, "six occurred in July, *seventeen* in August, *nineteen* in September, and five in October, making the whole number save one," which occurred in March.—Of the whole number of cases in the collection, 26 occurred during the summer of 1849, when epidemic cholera prevailed severely in that city. The dysentery was most prevalent as the cholera epidemic subsided.—In the summer of 1852 the cholera was again prevalent in Buffalo, but it was not accompanied or followed by dysentery as in 1849.

Of the 14 cases recorded by Dr. Flint previous to 1849, all of which were sporadic cases, *none* died. Of the 26 cases recorded during the summer of 1849, *four* died. These facts show in a clear light not merely the influence of the season of the year in determining the number of cases, but also the influence of epidemic agencies in determining their *fatality*. In the treatment of dysentery the writer seems to have limited himself chiefly to the use of calomel and opiates, with occasional astringents and laxatives. He divides the whole number of cases, (49,) into two groups. The one consisting of 23 cases, were all treated with calomel, or calomel and opiates internally, aided in some instances with opiate enemas. Of these 5 terminated fatally.

The other group, consisting of 26 cases, were all treated with opiates aided by anodyne and astringent enemas, without any calomel or mercurials in any form. Of these, 6 died. The average duration of the treatment in both groups, was very nearly the same. The practical inference to be drawn from these numerical statements of the author, is, that *calomel* is simply *useless* in the treatment of dysentery ; an inference which goes far to prove the fallacy of the mode of comparison, and indeed the faultiness of all merely numerical results when applied as rules of practice. If all diseases were caused by specific agents, and all remedies acted simply as antidotes, it would be easy to determine by numerical results the relative value of each article. But while dysentery, like most of the acute diseases, arises from a variety of causes and presents essentially different symptoms in successive seasons, and at different stages of its own progress, it will require for its successful treatment such a variety of means as will serve to fulfil accurately the indications presented by each individual case, and at each stage of its process. An attempt to determine the value of a remedy in the treatment of an acute disease by giving it in twenty or thirty cases successively, and continuing it in all stages of the disease, is just as absurd as it would be to put the same sized clothes on twenty successive babies and then attempt to make the same fit all the way up to adult life. I do not wish the reader to infer that Dr. Flint has pursued any such indiscriminate practice; and yet the two following cases come not far short of it:—

No. 3. On the evening of the day on which dysenteric discharges appeared, 4 grs. of calomel were prescribed, and the remedy continued in doses of grs. ii. every two hours, until 12 grains had been taken. The so-called bilious stools not being produced, another dose of grs. iv. was given. Copious green evacuations followed. To these succeeded profuse sero-sanguinolent discharges, and great prostation. The disease ended fatally on the third day after this treatment was pursued.

No. 4 At the commencement of the disease, calomel was given in doses of grs. ii, every four hours, with enemas of a solution of the sulphate of morphia and kreosote. Continued only for one day, and the sulphate of morphia substituted. On the seventh day, (symptoms becoming worse,) calomel again prescribed in doses of grs.i. ss., every four hours. On the eight and ninth days, improvement. On the tenth day (calomel continued) the discharges became sero-sanguinolent, the pulse rose rapidly in frequency, and the case ended fatally on the eleventh day.

Here is one case with no mention of anything but calomel from the beginning to the end, and yet from its rapid progress and the character of the discharges it was probably one of a highly congestive or *choleraic* character; and another in which calomel is given three or four days in succession in the advanced stage of the disease, ending only with the termination of the case on the eleventh day.—That such cases afford no evidence whatever in regard to the value of calomel in certain forms and stages of dysentery, every reflecting reader will perceive. Every experienced practitioner knows that dysentery varies exceedingly in phenomena, both local and general; and consequently that the indications for treatment are equally variable. This is fully illustrated by Dr. Flint's cases. Thus of 14 *sporadic* cases recorded by him between 1838 and 1849, none proved fatal. But of 37 epidemic cases recorded during the years 1849–50–51 & 52, *ten* terminated fatally. Now, to include all these cases in one category, give the same remedy in all, and then endeavor to estimate the value of the remedy by the numerical results, is to include as identical, things essentially diverse, and none other than fallacious results could be expected. What is called the "*numerical method*" of investigation may be applied to certain branches of medical inquiry; and when restricted to such branches it is of great value. But from the very nature of acute diseases—their variableness in different seasons, and the variable circumstances and conditions of the vital forces at different stages in the progress of the same case—this method cannot be applied to special *therapeutic* investigations. That there are certain cases of dysentery in which calomel, given in a proper manner in the early stage, is a very valuable auxiliary remedy, very few will doubt. That there are other cases in which it would positively do harm, I am quite certain. That it is generally useless if not positively injurious in the advanced stage of the disease, is equally true. The true and only method of investigation, then, is not to group all cases together and deduce numerical inferences, but to compare carefully the phenomena of each case, and at each stage of its progress, for the purpose of ascertaining the actual differences or variations, and the exact application of particular remedies in accordance with such changes. These criticisms are aimed not merely at the work of Dr. Flint, but at the method of investigation which he has adopted, and which many seem disposed to apply in a manner not calculated to lead to any beneficial results. After having extended these comments much farther than I intended, I will only add, that the little monograph before me, entitled "Flint's Clinical Report on Dysentery," is a valuable addition to our medical literature, and is worthy of a place in every physician's library. And moreover, I hope the industrious author will live to make many more such. N. S. D.

